# Constructing Co_3_O_4_/La_2_Ti_2_O_7_ p-n Heterojunction for the Enhancement of Photocatalytic Hydrogen Evolution

**DOI:** 10.3390/nano12101695

**Published:** 2022-05-16

**Authors:** Haodong Wen, Wenning Zhao, Xiuxun Han

**Affiliations:** Institute of Optoelectronic Materials and Devices, Faculty of Materials Metallurgy and Chemistry, Jiangxi University of Science and Technology, Ganzhou 341000, China; whdrb2016@163.com

**Keywords:** photocatalysis, hydrogen evolution, p-n heterojunction, La_2_Ti_2_O_7_, Co_3_O_4_

## Abstract

Layered perovskite-type semiconductor La_2_Ti_2_O_7_ has attracted lots of attention in photocatalytic hydrogen evolution, due to the suitable energy band position for water splitting, high specific surface area, and excellent physicochemical stability. However, the narrow light absorption range and the low separation efficiency of photogenerated carriers limit its photocatalytic activity. Herein, plate-like La_2_Ti_2_O_7_ with uniform crystal morphology was synthesized in molten NaCl salt. A p-n heterojunction was then constructed through the in situ hydrothermal growth of p-type Co_3_O_4_ nanoparticles on the surface of n-type plate-like La_2_Ti_2_O_7_. The effects of Co_3_O_4_ loading on photocatalytic hydrogen evolution performance were investigated in detail. The results demonstrate that composite Co_3_O_4_/La_2_Ti_2_O_7_ possesses much better photocatalytic activity than the pure component. The composite photocatalyst with 1 wt% Co_3_O_4_ exhibits the highest hydrogen evolution rate of 79.73 μmol·g^−1^·h^−1^ and a good cycling stability. The photoelectrochemistry characterizations illustrate that the improvement of photocatalytic activity is mainly attributed to both the enhanced light absorption from the Co_3_O_4_ ornament and the rapid separation of photogenerated electron-hole pairs driven by the built-in electric field close to the p-n heterojunction. The results may provide further insights into the design of high-efficiency La_2_Ti_2_O_7_-based heterojunctions for photocatalytic hydrogen evolution.

## 1. Introduction

Owing to the high energy density, pollution-free combustion and abundance of the raw materials, hydrogen is perceived to be one of the most potential substitutes for traditional fossil fuels. The development and utilization of hydrogen energy could effectively ease the energy crisis and environmental deterioration. As a secondary source of energy, hydrogen does not exist freely in nature and only can be obtained from other sources of energy [1]. Among the various energy sources that can be used to produce hydrogen, solar power is highly superior in the abundance, economy, safety and environmental protection. Semiconductor-based photocatalytic water splitting is an efficient way to directly transform solar radiation to hydrogen energy [2,3,4,5]. In recent years, on account of proper band edge position, specific surface area and physicochemical stability, La_2_Ti_2_O_7_ has been widely explored in the field of photocatalytic splitting of water and degradation of pollutants [6,7,8,9,10,11,12,13,14]. However, relatively wide band gap (~3.8 eV) and high recombination rate of photogenerated electron-hole pairs in pure La_2_Ti_2_O_7_ lead to insufficient charge generation and separation, and thus the poor photocatalytic activity [15]. Coupling a suitable narrow band-gap semiconductor with La_2_Ti_2_O_7_ to form a heterojunction is undoubtedly an effective solution. Composite systems including CdS/La_2_Ti_2_O_7_ [16], La_2_Ti_2_O_7_/LaCrO_3_ [17] and La_2_Ti_2_O_7_/ZnIn_2_S_4_ [18] all demonstrated better photocatalytic performances than their individual counterparts.

Co_3_O_4_ is a typical p-type semiconductor with narrow band gap (1.6–2.2 eV) [19,20]. It has a good charge transport capability, and the electrical resistivity is in the order of 10^3^ Ω∙cm under room temperature [21]. It presents high photochemical stability both in acid and alkaline environments [22,23]. Co_3_O_4_ itself shows a negligible photocatalytic hydrogen evolution activity. However, constructing p-n heterojunction with another n-type semiconductor, for instance, Co_3_O_4_/g-C_3_N_4_ [24], Co_3_O_4_/CeO_2_ [25] and Co_3_O_4_/TiO_2_ [26], will greatly promote the photocatalytic performance, which is mainly due to the enhanced separation of photogenerated carriers under the action of built-in electric field in p-n heterojunction [27]. In consideration of the band position of Co_3_O_4_ [26], it is quite appropriate to form type II p-n heterojunction with La_2_Ti_2_O_7_ [28]. In addition, a variety of Co_3_O_4_ morphologies, such as nanoparticle (0D), nanorod (1D), and nanosheet (2D) can be facilely obtained [29,30,31]. In the heterojunction constructed with nanoparticles and nanosheets, not only the efficient separation and transport of photogenerated carriers, but also the enough exposure of active sites, can be guaranteed.

In this work, Co_3_O_4_ nanoparticles were grown in situ on the surface of plate-like La_2_Ti_2_O_7_ by hydrothermal method, thereby constructing a Co_3_O_4_/La_2_Ti_2_O_7_ p-n heterojunction. The effects and related mechanisms of Co_3_O_4_ loading on the photocatalytic hydrogen evolution performance were investigated in detail. Furthermore, the separation and transport behavior of photogenerated carriers in the Co_3_O_4_/La_2_Ti_2_O_7_ heterojunction was elucidated.

## 2. Materials and Methods

La_2_Ti_2_O_7_ was synthesized via molten salt method. Lanthanum oxide (La_2_O_3_, 99.99%, Macklin; Shanghai, China) and titanium dioxide (TiO_2_, P25, Macklin; Shanghai, China) were the starting materials, and sodium chloride (NaCl, 99.99%, Aladdin; Shanghai, China) served as the fluxing agent. All the chemicals were used as received. Typically, La_2_O_3_, TiO_2_ and NaCl powders were mixed with a molar ratio of 1:2:10, and then thoroughly ground in an agate mortar for 1 h. Subsequently, the mixture was placed in an alumina crucible and heated to 1150 °C in a muffle furnace (SX-G03163; Zhonghuan; Tianjin, China) for 7 h. After naturally cooling down to room temperature, the products were washed four times with hot deionized water to remove the fluxing agent. Finally, the powders were dried at 80 °C for 6 h to obtain the white plate-like La_2_Ti_2_O_7_.

Co_3_O_4_ nanoparticles were grown in situ on the surface of La_2_Ti_2_O_7_ according to a previously reported hydrothermal method [26]. Then, 100 mg of plate-like La_2_Ti_2_O_7_ was added into 60 mL NaOH solution (0.1 M), and dispersed in an ultrasonic bath for 10 min. Then, 5 mg of hexadecyl trimethyl ammonium bromide (CTAB, 99%, Rhawn; Shanghai, China) and a certain amount of cobaltous nitrate hexahydrate (Co(NO_3_)_2_·6H_2_O, 99.99%, Aladdin; Shanghai, China) were added into solution, followed by stirring for 30 min. The mixture was sealed in a Teflon-lined stainless-steel autoclave and kept reacting at 110 °C for 24 h. The obtained product was alternately washed with deionized water and ethanol, as well as dried at 80°C to gain the final composite. For the convenience of following discussions, the loading amounts of Co_3_O_4_ on La_2_Ti_2_O_7_ (weight ratio) of 0.25%, 0.50%, 1.00%, 2.00%, and 5.00% are marked as LC1, LC2, LC3, LC4, and LC5, respectively. Besides, for comparison, Co_3_O_4_ nanoparticles were prepared under identical conditions without the addition of La_2_Ti_2_O_7_ powder.

X-ray diffraction (XRD) patterns of all samples were measured using Tongda TD3700 (Dandong, China) X-ray diffractometer. Scanning electron microscopy (SEM) images as well as energy dispersive X-ray spectrum (EDS) were acquired from Phenom Pro (Eindhoven, Netherlands) and JEOL JSM-6701F microscopes (Tokyo, Japan). Transmission electron microscopy (TEM) images were obtained from FEI TalosF200x microscope (Eindhoven, Netherlands). Thermo Scientific K-Alpha spectrometer (Waltham, MA, USA) with an Al Kα X-ray source was employed to record the X-ray photoelectron spectrum (XPS). All of the binding energies were calibrated by the C 1s peak at 284.80 eV. The diffuse reflectance spectrum measurements were taken with Shimadzu UV-2600 UV-Vis spectrophotometer (Kyoto, Japan). The photoluminescence spectra were characterized by Horiba FluoroMax-4 fluorescence spectrometer (Edison, NJ, USA) with an excitation wavelength of 340 nm.

All photocatalytic tests were performed on a Perfectlight Labsolar-IIIAG on-line photocatalytic analysis system (Beijing, China). Typically, 50 mg photocatalyst was dispersed into 10 vol% methanol aqueous solution (100 mL) and underwent an ultrasonic treatment for 10 min. Then, the reactant solution was transferred into a quartz reactor and connected to the analysis system. The system was vacuumized for 30 min to completely deair prior to light irradiation. The temperature of reactant solution was kept at 5 °C during the whole testing process. A 300 W xenon lamp (Solar-500, NBET; Beijing, China) was used to supply UV-Vis light. The produced H_2_ was analyzed by a gas chromatography (GC7900, techcomp; Shanghai, China) equipped with a thermal conductive detector. High-purity Ar was applied as the carrier gas. For the stability test, the catalyst was collected and recycled at intervals of 5 h.

The photoelectrochemical measurements including photocurrent response, Nyquist plot and Mott-Schottky curve, were carried out using an electrochemical workstation (CHI660E, Chen Hua; Shanghai, China). The preparation of the working electrode was conducted prior to the measurements. In short, 10 mg photocatalyst was dispersed into a mixed solvent containing 1 mL ethanol and 10 μL Nafion solution (5 wt%, Dupont; Wilmington, DE, USA), followed by ultrasonic treatment for 1 h to form a homogeneous solution. The electrode was formed by drop-coating mixed solution onto the cleaned FTO glass (Opvtech; Yingkou, China) (3 × 2 cm^2^), and an active surface area of ~1 cm^2^ was delimited by nonconductive epoxy. A standard three-electrode cell system was adopted during measurement. Ag/AgCl electrode, Pt plate, and 0.1 M Na_2_SO_4_ aqueous solution were employed as reference electrode, counter electrode, and electrolyte, respectively. The electrolyte was purged with Ar gas for 30 min before all measurements. Photocurrent response was measured at a bias voltage of 0.2 V (vs. Ag/AgCl) with the irradiation of 300 W xenon lamp (Solar-500, NBET; Beijing, China). Nyquist plot test was conducted with an amplitude of 5 mV, under the open circuit voltage. The frequency ranged from 10^−1^ to 10^5^ Hz. 

## 3. Results and Discussion

**Figure 1:**
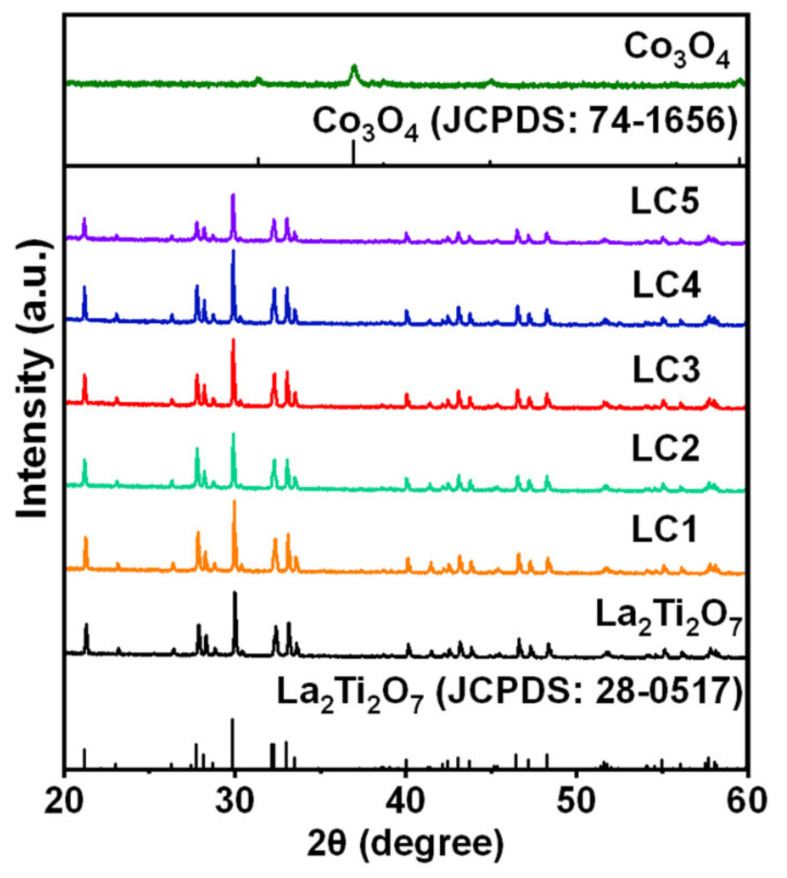
XRD patterns of La_2_Ti_2_O_7_, Co_3_O_4_ and Co_3_O_4_/La_2_Ti_2_O_7_ composites.

The prepared La_2_Ti_2_O_7_, Co_3_O_4_ and composite materials were examined by XRD, and the corresponding patterns are depicted in Figure 1. As can been seen, all diffraction peaks of the La_2_Ti_2_O_7_ sample agree well with the standard profile (JCPDS: 28-0517) [32]. For the pure Co_3_O_4_ sample, five weak diffraction peaks at 19.12°, 31.26°, 36.98°, 44.96°, and 59.52°, respectively, corresponding to (111), (220), (311), (400), and (511) crystallographic planes of standard profile (JCPDS: 74-1656) [33], are barely observed. The weak intensity and large width of diffraction peak indicate the low crystallinity and small size of Co_3_O_4_. The diffraction peaks of Co_3_O_4_/La_2_Ti_2_O_7_ composites are basically consistent with those of the pure La_2_Ti_2_O_7_ sample. No diffraction peak of Co_3_O_4_ can be detected. This is due to the small size and low loading amount of Co_3_O_4_. However, when the weight ratio of Co_3_O_4_/La_2_Ti_2_O_7_ reaches 5.00% (LC5), the reduction of diffraction peak intensity occurs. This may be ascribed to the enhanced coverage of Co_3_O_4_ on the surface of La_2_Ti_2_O_7_. Similar phenomena were commonly observed for composite catalysts [34].

**Figure 2:**
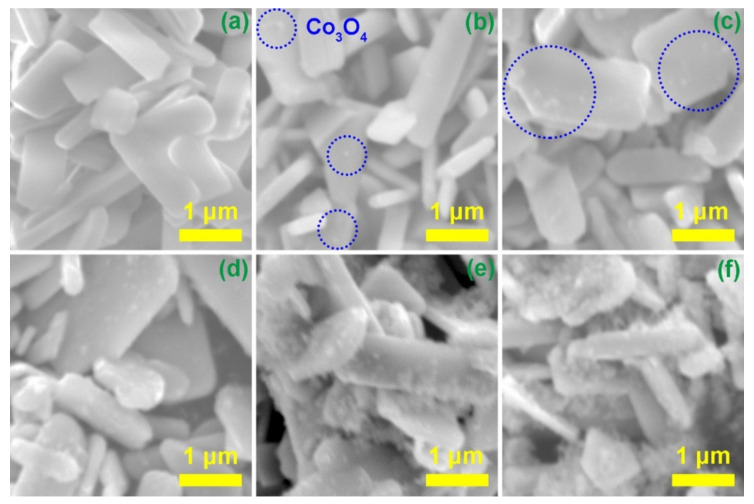
SEM images of (**a**) La_2_Ti_2_O_7_, (**b**) LC1, (**c**) LC2, (**d**) LC3, (**e**) LC4, and (**f**) LC5.

**Figure 3:**
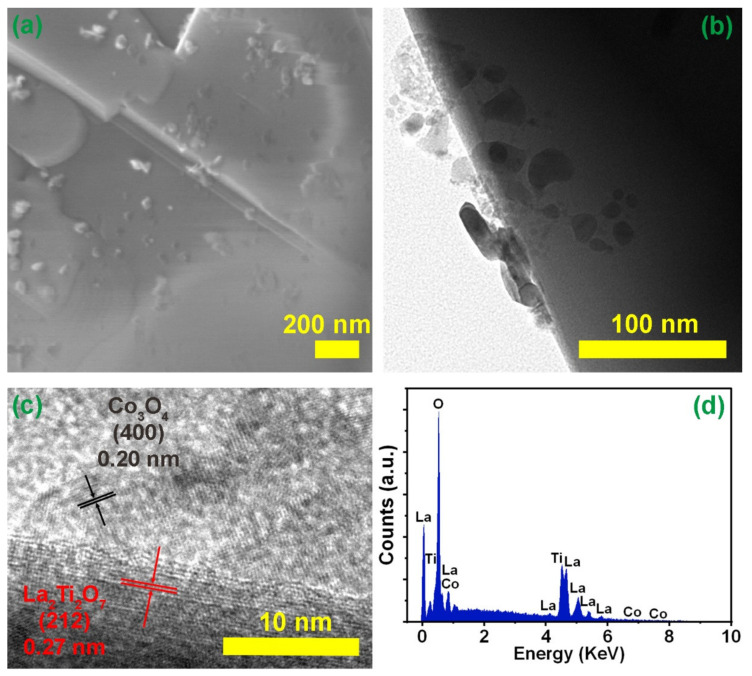
(**a**) High-resolution SEM image of LC3; (**b**) TEM image of LC3; (**c**) HR-TEM image of LC3; (**d**) EDS spectrum of LC3.

The overall morphologies of prepared pure La_2_Ti_2_O_7_ and different composites were characterized by SEM. As shown in Figure 2a, the La_2_Ti_2_O_7_ sample exhibits a plate-like morphology and smooth surface. The horizontal average size is about 2 μm. In the composite samples, nanoparticles emerge on the smooth surface of La_2_Ti_2_O_7_, and the number of nanoparticles grows with the increase of the loading amount of Co_3_O_4_, as displayed in Figure 2b–f. When the loading amount increases to 5.00% (LC5), most of the surface of La_2_Ti_2_O_7_ is covered by nanoparticles. As it can been seen from the high-resolution image of LC3 (Figure 3a), nanoparticles distribute on the surface of La_2_Ti_2_O_7_. It also demonstrates that the hydrothermal process did not change the structure and morphology of La_2_Ti_2_O_7_. To analyze the elemental composition of composite, EDS measurement was carried out. As shown in Figure 3d, the La, Ti, O, and Co elements are all detected. It implies what on the surface of La_2_Ti_2_O_7_ are most likely Co_3_O_4_ nanoparticles. Figure 3b display the TEM images of sample LC3. It can be seen that Co_3_O_4_ nanoparticles with irregular shapes were grown on the surface of La_2_Ti_2_O_7_. Both the (212) crystallographic plane of La_2_Ti_2_O_7_ [35] and the (400) crystallographic plane of Co_3_O_4_ [36] can be discerned (Figure 3c). It indicates that a heterojunction with a close contact is formed between La_2_Ti_2_O_7_ and Co_3_O_4_.

**Figure 4:**
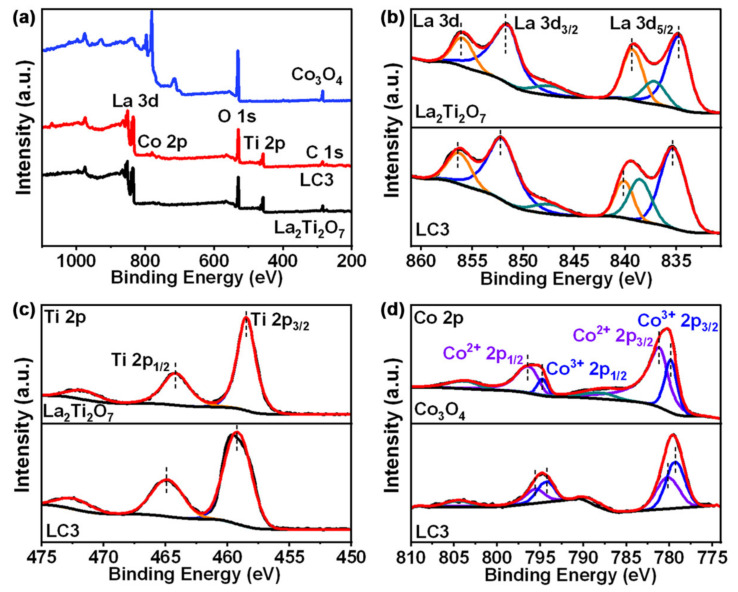
(**a**) XPS survey spectra of La_2_Ti_2_O_7_, Co_3_O_4_ and LC3. High-resolution XPS spectra of (**b**) La 3d, (**c**) Ti 2p, and (**d**) Co 2p.

To analyze the chemical state of the Co_3_O_4_/La_2_Ti_2_O_7_ composite and the formation of heterojunction, the XPS spectra of La_2_Ti_2_O_7_, Co_3_O_4_, and composite (LC3) were measured and exhibited in Figure 4. The XPS survey spectrum of LC3 (Figure 4a) manifests the binding energy peaks of La 3d, Co 2p, O 1s, Ti 2p, and C 1s, identifying the coexistence of all elements from Co_3_O_4_ and La_2_Ti_2_O_7_ in the composite sample. The C 1s peaks in all samples originate from the contaminating carbon in the test [37]. Figure 4b–d give the high-resolution XPS spectra of La 3d, Ti 2p, and Co 2p, respectively. For pure La_2_Ti_2_O_7_, the binding energy peaks located at 834.75 eV and 839.34 eV correspond to La 3d_5/2_, while the peaks at 851.75 eV and 856.11 eV belong to La 3d_3/2_ (Figure 4b) [38]. The peaks of 458.50 eV and 464.21 eV are assigned to Ti 2p_3/2_ and Ti 2p_1/2_, respectively (Figure 4c) [8]. For pure Co_3_O_4_, the peaks at 779.87 eV and 794.77 eV can be ascribed to Co^3+^ 2p_3/2_ and Co^3+^ 2p_1/2_, respectively (Figure 4c). Meanwhile, the peaks of 781.23 eV and 796.49 eV can be attributed to Co^2+^ 2p_3/2_ and Co^2+^ 2p_1/2_, respectively [39]. As for Co_3_O_4_/La_2_Ti_2_O_7_ composite (LC3), obviously, all peaks of La 3d (835.36 eV, 840.17 eV, 852.24 eV, and 856.42 eV) as well as Ti 2p (459.26 eV and 464.91 eV) shift towards the direction of high binding energy (Figure 4b,c). On the contrary, all peaks of Co 2p (779.31 eV, 794.22 eV, 780.20 eV, and 795.58 eV) move towards the direction of low binding energy. It reveals the strong electronic interactions between Co_3_O_4_ and La_2_Ti_2_O_7_ in the composite: part of the electrons transfer from n-type La_2_Ti_2_O_7_ to p-type Co_3_O_4_, leading to different electronic behaviors in the two materials. Moreover, it further proved that a heterojunction with a close contact is formed [40].

**Figure 5:**
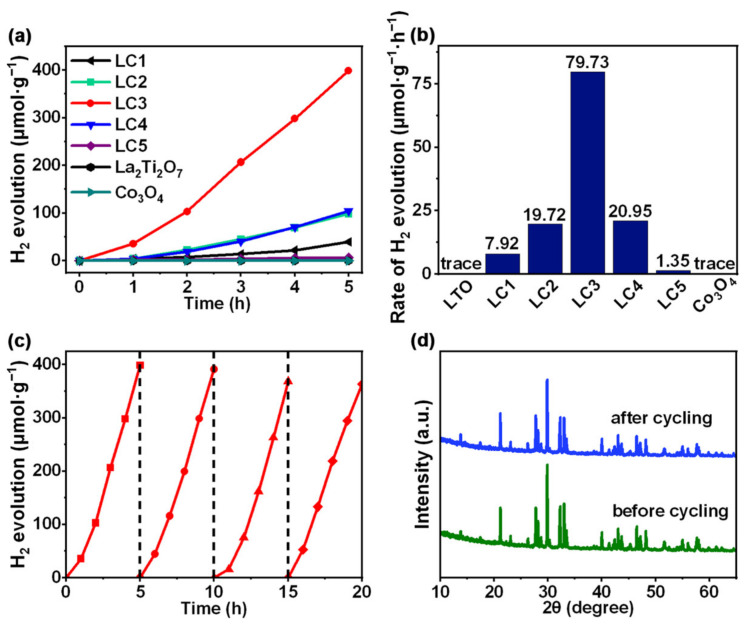
(**a**) Time courses of photocatalytic H_2_ evolution of La_2_Ti_2_O_7_, Co_3_O_4_, and composite; (**b**) H_2_ evolution rates of La_2_Ti_2_O_7_ (LTO), Co_3_O_4_ and composite; (**c**) Cycling experiment of H_2_ evolution for LC3; (**d**) XRD patterns of LC3 before and after the cycling test.

The photocatalytic hydrogen evolution activities of La_2_Ti_2_O_7_, Co_3_O_4_ and composite samples were evaluated under UV-Vis light irradiation in methanol aqueous solution, and the results are depicted in Figure 5a,b. Both pure La_2_Ti_2_O_7_ and pure Co_3_O_4_ present negligible amounts of hydrogen evolution. Instead of participating in the hydrogen evolution reaction, most of the photogenerated electrons in La_2_Ti_2_O_7_ and Co_3_O_4_ lose due to the recombination. After loading of Co_3_O_4_ nanoparticles, the photocatalytic hydrogen evolution rates of La_2_Ti_2_O_7_ composites are remarkably improved and tends to elevate with the increase of loading amount, until reaching the highest value of 79.73 μmol·g^−1^·h^−1^ at the Co_3_O_4_ loading amount of 1.00%. However, a further increase of the loading amount leads to a decline in the hydrogen evolution rate. When the Co_3_O_4_ loading amount expands to 5.00% (LC5), the hydrogen evolution rate dramatically drops to 1.35 μmol·g^−1^·h^−1^, presumably due to fact that the La_2_Ti_2_O_7_ surface is unduly covered by Co_3_O_4_, resulting in the limited exposure of active sites for hydrogen evolution, as can been seen from the SEM image (Figure 2f). Furthermore, the best performing Co_3_O_4_/La_2_Ti_2_O_7_ was subjected to four consecutive cycles of photocatalytic hydrogen evolution to examine the catalyst stability and durability. Sample LC3 displays no significant reduction of the hydrogen evolution rate after multiple reaction cycles (20 h), depicting an excellent photocatalytic activity (Figure 5c). The slight decrease is supposed to originate from the photocatalyst loss in the process of collection. The catalyst was reexamined by XRD spectra after the circling reactions. As shown in Figure 5d, no difference can be detected from the XRD patterns of the sample before and after the cycling test, which further confirms the high stability of the Co_3_O_4_/La_2_Ti_2_O_7_ composite photocatalyst.

**Figure 6:**
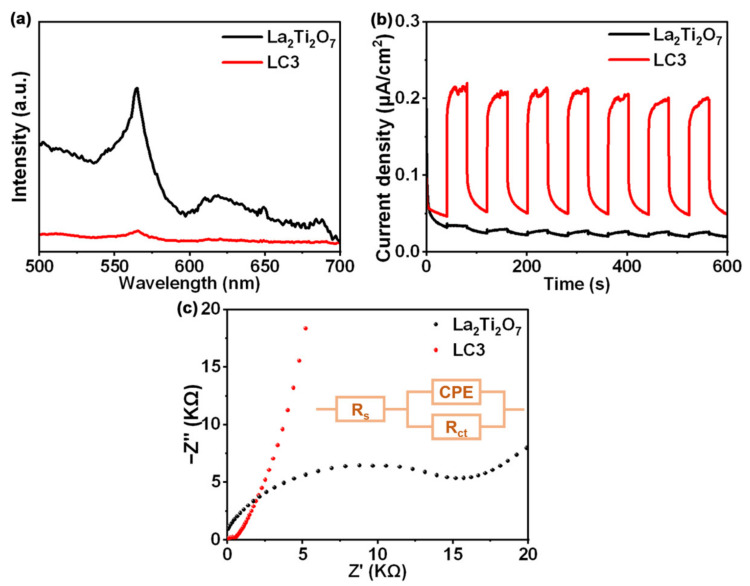
(**a**) Photoluminescence spectra La_2_Ti_2_O_7_ and LC3; (**b**) Photocurrent responses of La_2_Ti_2_O_7_ and LC3; (**c**) Nyquist plots of La_2_Ti_2_O_7_ and LC3.

Figure 6 shows the photoluminescence spectra, the photocurrent responses, as well as the Nyquist plots of pure La_2_Ti_2_O_7_ and LC3 photocatalysts. The photoluminescence intensity of a semiconductor closely correlates with the recombination rate of photogenerated carriers. As can be seen from Figure 6a, the photoluminescence intensity of sample LC3 is much lower than that of single La_2_Ti_2_O_7_, implying that the loading of Co_3_O_4_ prompts the separation of photogenerated carriers and thus reduces the radiative recombination rate to some extent [18]. Besides, sample LC3 presents a higher photocurrent density than La_2_Ti_2_O_7_ (Figure 6b), well indicating the effective extraction of photogenerated carriers via the formation Co_3_O_4_/La_2_Ti_2_O_7_ heterojunction [41]. The feeble photocurrent density of La_2_Ti_2_O_7_ illustrates a serious carrier loss in pure La_2_Ti_2_O_7_. Furthermore, electrochemical impedance spectrum (EIS) was utilized to investigate the interfacial charge transport property, and the Nyquist plots is presented in Figure 6c. Through equivalent circuit fitting, the charge transfer resistance (*R*_ct_) values of La_2_Ti_2_O_7_ and LC3 are estimated to be 19.2 and 0.95 kΩ, respectively. It means that the composite has a lower charge transfer resistance and the accelerated charge migration. The above discussions prove that a heterojunction was constructed between Co_3_O_4_ nanoparticles and plate-like La_2_Ti_2_O_7_, and an efficient interface channel for both charge separation and transport was established. As a result, the spatial separation between electrons and holes suppresses the carrier recombination and improves their capabilities to participate in the hydrogen evolution reaction.

**Figure 7:**
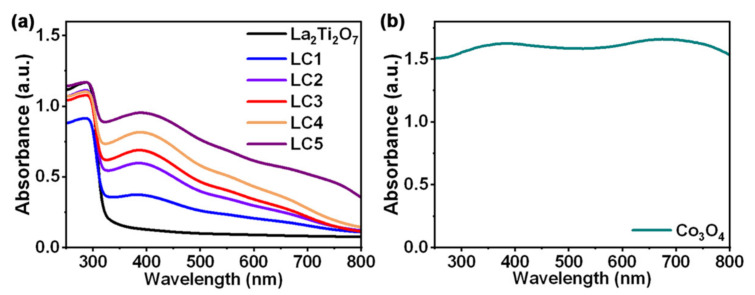
(**a**) UV-Vis absorption spectra of La_2_Ti_2_O_7_ and composites; (**b**) UV-Vis absorption spectrum of Co_3_O_4_.

The optical absorption properties of the samples were examined by diffuse reflectance spectroscopy in the range of 250 to 800 nm. The corresponding UV-Vis absorption spectra are depicted in Figure 7. La_2_Ti_2_O_7_ has a strong absorption in the wavelength region lower than 320 nm, while Co_3_O_4_ exhibits a strong absorption in the whole UV-Vis range. The loading of Co_3_O_4_ nanoparticles onto La_2_Ti_2_O_7_ highly enhances the light absorption capability within the visible range. It is thus beneficial to generate more carriers and prompt the photocatalytic hydrogen evolution activity.

**Figure 8:**
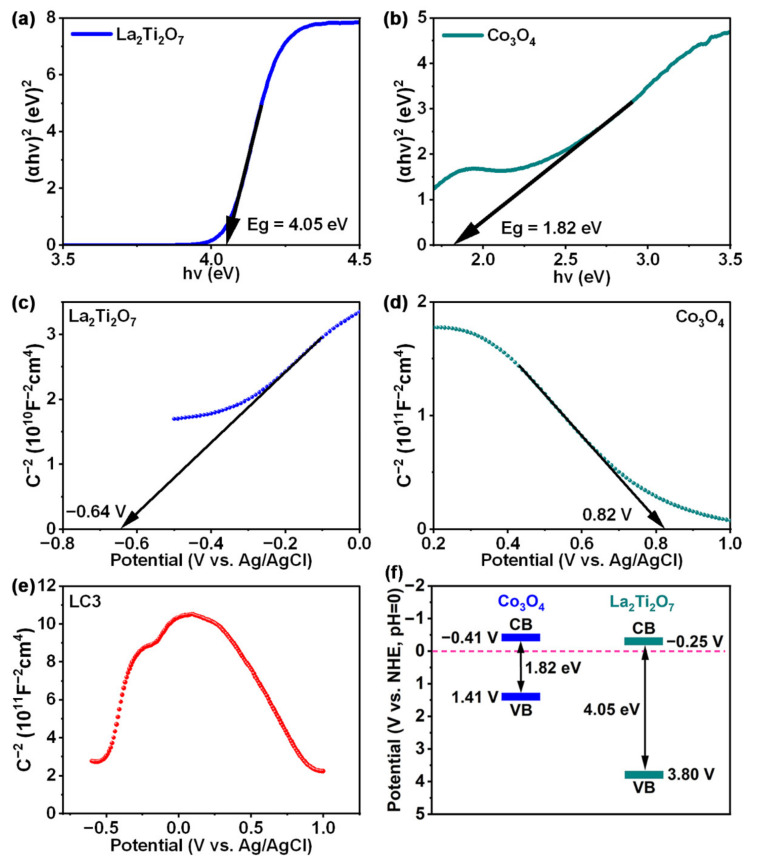
(**a**) Tauc plot of La_2_Ti_2_O_7_; (**b**) Tauc plot of Co_3_O_4_; (**c**) Mott-Schottky curve of La_2_Ti_2_O_7_; (**d**) Mott-Schottky curve of Co_3_O_4_; (**e**) Mott-Schottky curve of LC3; (**f**) Schematic diagram of energy band structure of Co_3_O_4_ and La_2_Ti_2_O_7_.

The optical band gap of Co_3_O_4_ and La_2_Ti_2_O_7_ were determined to be 1.82 eV and 4.05 eV by linear fitting of Tauc plots, respectively, as displayed in Figure 8a,b. In order to figure out the energy band positions of Co_3_O_4_ and La_2_Ti_2_O_7_, Mott-Schottky analysis was performed. As demonstrated in Figure 8c,d, the negative slope of Mott-Schottky curve for Co_3_O_4_ and the positive one for La_2_Ti_2_O_7_ indicate that Co_3_O_4_ and La_2_Ti_2_O_7_ are p-type and n-type semiconductors, respectively. Additionally, the flat-band potentials (*E*_fb_) of Co_3_O_4_ and La_2_Ti_2_O_7_ are deduced to be 0.82 V and −0.64 V (vs. Ag/AgCl, pH = 7) by the extrapolation of apparent slopes. After potential conversion following the equation *E*_fb_ (vs. NHE, pH = 0) = *E*_fb_ (vs. Ag/AgCl, pH = 7) + 0.059 × pH + 0.179 [26], the *E*_fb_ values of Co_3_O_4_ and La_2_Ti_2_O_7_ are calculated to be 1.41 V and −0.05 V (vs. NHE, pH = 0), respectively. Based on the deduced bandgap of 1.82 eV, the conduction band minimum (CBM) of Co_3_O_4_ can be calculated to be −0.41 V. Considering that the CBM of an n-type semiconductor is generally 0.2 V higher than the *E*_fb_ [42], the CBM and valence band maximum (VBM) values of La_2_Ti_2_O_7_ can be figured to be −0.25 eV and 3.80 eV, respectively. Furthermore, the Mott-Schottky curve of composite sample LC3 is measured and depicted in Figure 8e. The inverted V-shaped curve confirms that the p-n heterojunction is successfully constructed [43,44,45]. Figure 8f shows the schematic diagram of the energy band structure of Co_3_O_4_ and La_2_Ti_2_O_7_. Obviously, both the CBM and VBM of p-type Co_3_O_4_ are more negative than those of n-type La_2_Ti_2_O_7_.

**Figure 9:**
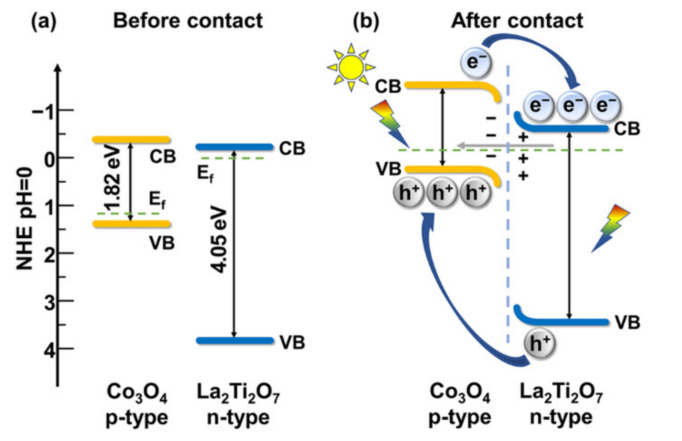
Band energy alignments of the composite (**a**) before contact and (**b**) after contact.

Once Co_3_O_4_ and La_2_Ti_2_O_7_ are connected together, a p-n heterojunction is formed. Since there are more electrons in the n-type La_2_Ti_2_O_7_ than in the p-type Co_3_O_4_, electrons diffuse from the n-side to p-side after the two-material connection. Similarly, holes diffuse from the p-side to the n-side of the heterojunction. Consequently, the diffusing away of carriers from the near vicinity of the junction leaves behind ionized dopants, which establishes a space charge region (depletion layer) near the junction interface. The unbalanced charges at each side of the junction results in the formation of a built-in electric field with the direction from La_2_Ti_2_O_7_ to Co_3_O_4_. The electric field induces a downward band bending in Co_3_O_4_ and an upward band bending in La_2_Ti_2_O_7_, as depicted in Figure 9.

Under the light irradiation, both La_2_Ti_2_O_7_ and Co_3_O_4_ can be excited, generating electron-hole pairs. In the space charge region, electrons and holes are driven towards the opposite direction by the built-in electric field, thereby achieving the spatial separation. Subsequently, the photogenerated electrons enter n-type La_2_Ti_2_O_7_, and participate in the water reduction reaction. In a similar way, photogenerated holes pass through the p-type Co_3_O_4_, and participate in oxidation reaction. Moreover, in the vicinity of the depletion layer, the photogenerated carriers with a lifetime long enough to diffuse into the space charge region can also be collected. Hence, the p-n heterojunction constructed by Co_3_O_4_ nanoparticle and plate-like La_2_Ti_2_O_7_ effectively promotes the separation and transport of photogenerated carriers, thereby remarkably improving the photocatalytic hydrogen production activity.

## 4. Conclusions

In summary, Co_3_O_4_ nanoparticles were grown in situ on the surface of plate-like La_2_Ti_2_O_7_ by hydrothermal method, and a Co_3_O_4_/La_2_Ti_2_O_7_ p-n heterojunction was constructed. The hydrogen evolution performance of La_2_Ti_2_O_7_ loaded with Co_3_O_4_ nanoparticles is significantly enhanced as compared with pure La_2_Ti_2_O_7_. The highest hydrogen evolution rate of 79.73 μmol·g^−1^·h^−1^ occurs for the sample loaded with 1 wt% Co_3_O_4_. Moreover, the composite catalyst exhibits an excellent cycling stability. The improvement of hydrogen evolution performance is ascribed to the enhanced light absorption and accelerated carrier separation/transfer via the built-in electric field after loading Co_3_O_4_ with proper amount on La_2_Ti_2_O_7_. The work raises a facile strategy to realized efficient photocatalytic hydrogen evolution through La_2_Ti_2_O_7_-based heterojunction without involving noble metals.

## Data Availability

The data presented in this study are available on request from the corresponding author.
